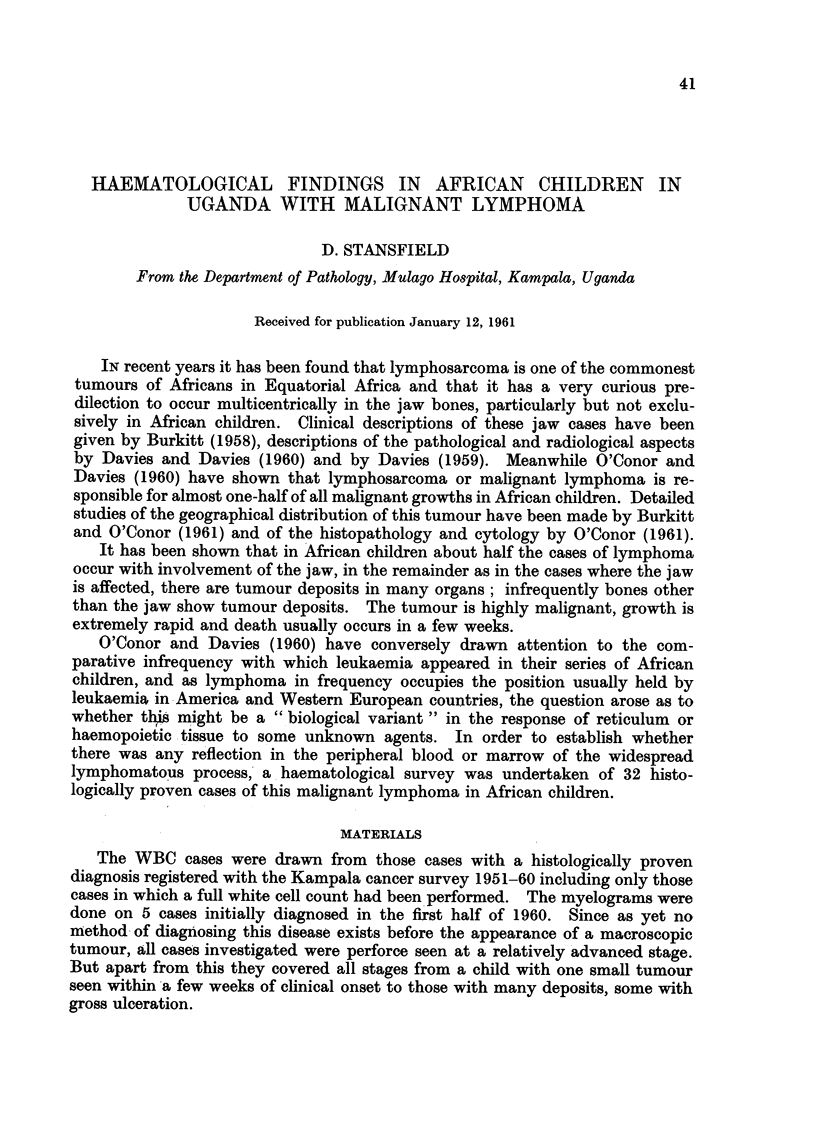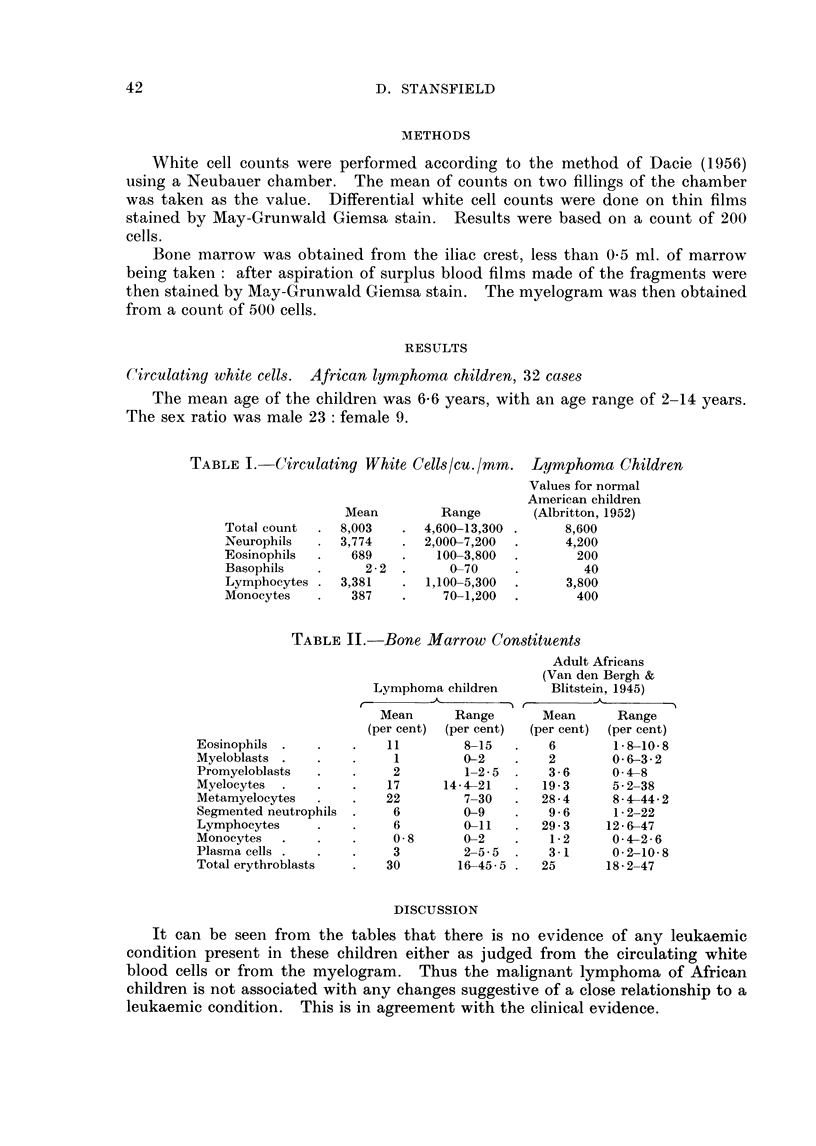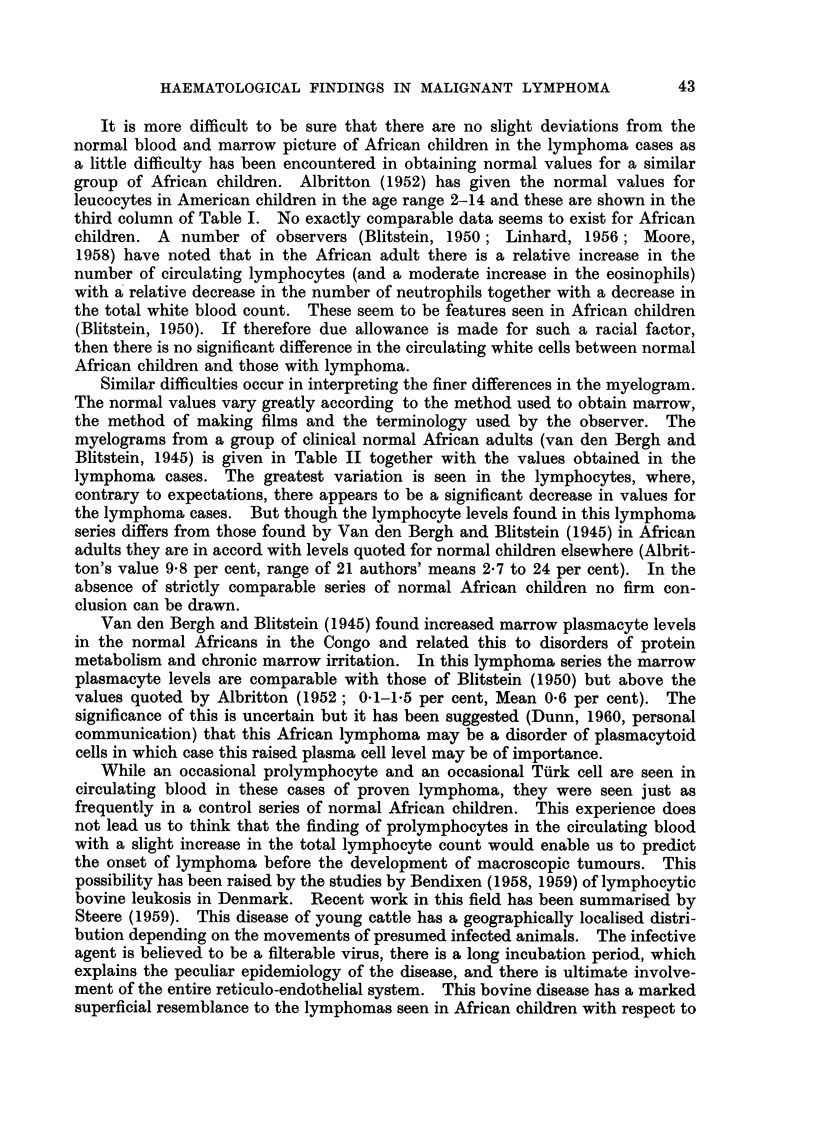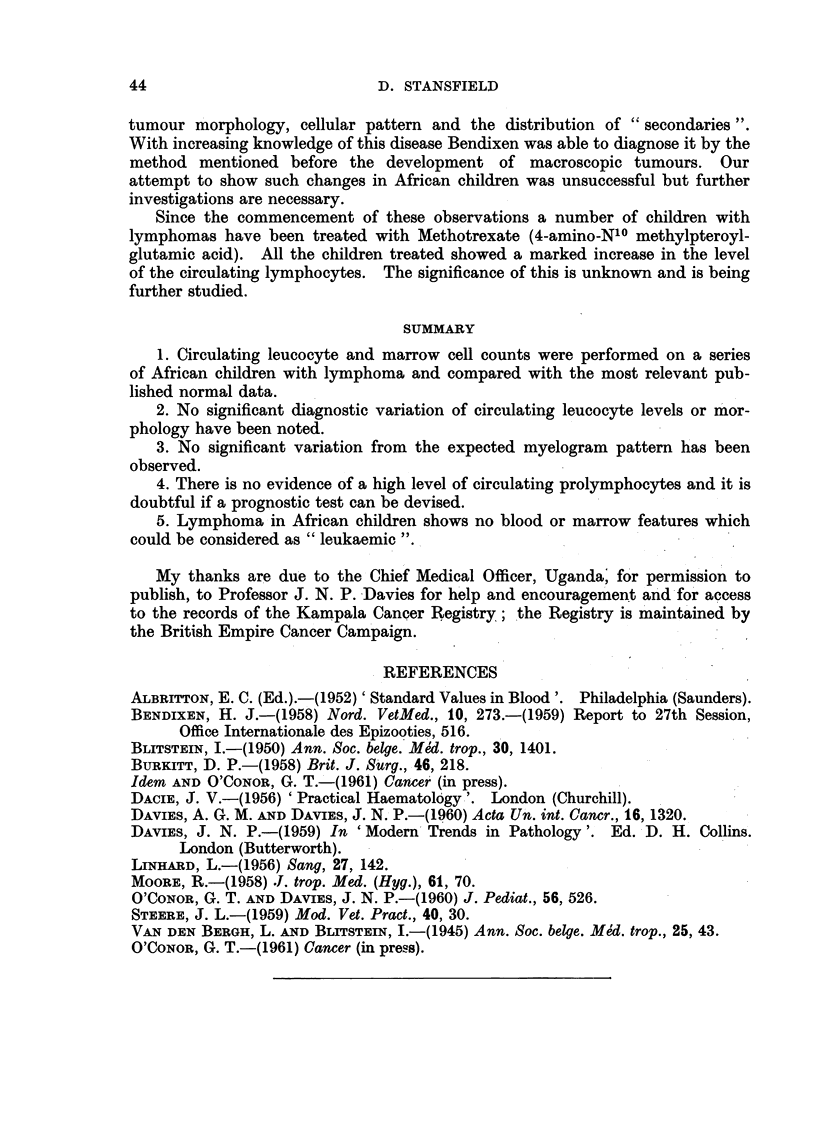# Haematological Findings in African Children in Uganda with Malignant Lymphoma

**DOI:** 10.1038/bjc.1961.5

**Published:** 1961-03

**Authors:** D. Stansfield


### HAEMATOLOGICAL FINDINGS IN AFRICAN CHILDREN IN UGANDA WITH MALIGNANT LYMPHOMA

D. STANSFIELD

*From the Department of Pathology, Mulago Hospital, Kampala, Uganda*

Received for publication January 12, 1961

IN recent years it has been found that lymphosarcoma is one of the commonest tumours of Africans in Equatorial Africa and that it has a very curious predilection to occur multicentrically in the jaw bones, particularly but not exclusively in African children. Clinical descriptions of these jaw cases have been given by Burkitt (1958), descriptions of the pathological and radiological aspects by Davies and Davies (1960) and by Davies (1959). Meanwhile O'Conor and Davies (1960) have shown that lymphosarcoma or malignant lymphoma is responsible for almost one-half of all malignant growths in African children. Detailed studies of the geographical distribution of this tumour have been made by Burkitt and O'Conor (1961) and of the histopathology and cytology by O'Conor (1961).

It has been shown that in African children about half the cases of lymphoma occur with involvement of the jaw, in the remainder as in the cases where the jaw is affected, there are tumour deposits in many organs ; infrequently bones other than the jaw show tumour deposits. The tumour is highly malignant, growth is extremely rapid and death usually occurs in a few weeks.

O'Conor and Davies (1960) have conversely drawn attention to the comparative infrequency with which leukaemia appeared in their series of African children, and as lymphoma in frequency occupies the position usually held by leukaemia in America and Western European countries, the question arose as to whether this might be a " biological variant " in the response of reticulum or haemopoietic tissue to some unknown agents. In order to establish whether there was any reflection in the peripheral blood or marrow of the widespread lymphomatous process, a haematological survey was undertaken of 32 histologically proven cases of this malignant lymphoma in African children.

### MATERIALS

The WBC cases were drawn from those cases with a histologically proven diagnosis registered with the Kampala cancer survey 1951–60 including only those cases in which a full white cell count had been performed. The myelograms were done on 5 cases initially diagnosed in the first half of 1960. Since as yet no method of diagnosing this disease exists before the appearance of a macroscopic tumour, all cases investigated were perforce seen at a relatively advanced stage. But apart from this they covered all stages from a child with one small tumour seen within a few weeks of clinical onset to those with many deposits, some with gross ulceration.

### METHODS

White cell counts were performed according to the method of Dacie (1956) using a Neubauer chamber. The mean of counts on two fillings of the chamber was taken as the value. Differential white cell counts were done on thin films stained by May-Grunwald Giemsa stain. Results were based on a count of 200 cells.

Bone marrow was obtained from the iliac crest, less than 0·5 ml. of marrow being taken : after aspiration of surplus blood films made of the fragments were then stained by May-Grunwald Giemsa stain. The myelogram was then obtained from a count of 500 cells.

### RESULTS

*Circulating white cells. African lymphoma children,* 32 *cases*

The mean age of the children was 6·6 years, with an age range of 2–14 years. The sex ratio was male 23 : female 9.

TABLE I.—*Circulating White Cells/cu./mm. Lymphoma Children*

| | Mean | Range | Values for normal American children (Albritton, 1952) |
|---|---|---|---|
| Total count | 8,003 | 4,600–13,300 | 8,600 |
| Neurophils | 3,774 | 2,000–7,200 | 4,200 |
| Eosinophils | 689 | 100–3,800 | 200 |
| Basophils | 2·2 | 0–70 | 40 |
| Lymphocytes | 3,381 | 1,100–5,300 | 3,800 |
| Monocytes | 387 | 70–1,200 | 400 |

TABLE II.—*Bone Marrow Constituents*

| | Lymphoma children | | Adult Africans (Van den Bergh & Blitstein, 1945) | |
|---|---|---|---|---|
| | Mean (per cent) | Range (per cent) | Mean (per cent) | Range (per cent) |
| Eosinophils | 11 | 8–15 | 6 | 1·8–10·8 |
| Myeloblasts | 1 | 0–2 | 2 | 0·6–3·2 |
| Promyeloblasts | 2 | 1–2·5 | 3·6 | 0·4–8 |
| Myelocytes | 17 | 14·4–21 | 19·3 | 5·2–38 |
| Metamyelocytes | 22 | 7–30 | 28·4 | 8·4–44·2 |
| Segmented neutrophils | 6 | 0–9 | 9·6 | 1·2–22 |
| Lymphocytes | 6 | 0–11 | 29·3 | 12·6–47 |
| Monocytes | 0·8 | 0–2 | 1·2 | 0·4–2·6 |
| Plasma cells | 3 | 2–5·5 | 3·1 | 0·2–10·8 |
| Total erythroblasts | 30 | 16–45·5 | 25 | 18·2–47 |

### DISCUSSION

It can be seen from the tables that there is no evidence of any leukaemic condition present in these children either as judged from the circulating white blood cells or from the myelogram. Thus the malignant lymphoma of African children is not associated with any changes suggestive of a close relationship to a leukaemic condition. This is in agreement with the clinical evidence.

It is more difficult to be sure that there are no slight deviations from the normal blood and marrow picture of African children in the lymphoma cases as a little difficulty has been encountered in obtaining normal values for a similar group of African children. Albritton (1952) has given the normal values for leucocytes in American children in the age range 2–14 and these are shown in the third column of Table I. No exactly comparable data seems to exist for African children. A number of observers (Blitstein, 1950; Linhard, 1956; Moore, 1958) have noted that in the African adult there is a relative increase in the number of circulating lymphocytes (and a moderate increase in the eosinophils) with a relative decrease in the number of neutrophils together with a decrease in the total white blood count. These seem to be features seen in African children (Blitstein, 1950). If therefore due allowance is made for such a racial factor, then there is no significant difference in the circulating white cells between normal African children and those with lymphoma.

Similar difficulties occur in interpreting the finer differences in the myelogram. The normal values vary greatly according to the method used to obtain marrow, the method of making films and the terminology used by the observer. The myelograms from a group of clinical normal African adults (van den Bergh and Blitstein, 1945) is given in Table II together with the values obtained in the lymphoma cases. The greatest variation is seen in the lymphocytes, where, contrary to expectations, there appears to be a significant decrease in values for the lymphoma cases. But though the lymphocyte levels found in this lymphoma series differs from those found by Van den Bergh and Blitstein (1945) in African adults they are in accord with levels quoted for normal children elsewhere (Albritton's value 9·8 per cent, range of 21 authors' means 2·7 to 24 per cent). In the absence of strictly comparable series of normal African children no firm conclusion can be drawn.

Van den Bergh and Blitstein (1945) found increased marrow plasmacyte levels in the normal Africans in the Congo and related this to disorders of protein metabolism and chronic marrow irritation. In this lymphoma series the marrow plasmacyte levels are comparable with those of Blitstein (1950) but above the values quoted by Albritton (1952; 0·1–1·5 per cent, Mean 0·6 per cent). The significance of this is uncertain but it has been suggested (Dunn, 1960, personal communication) that this African lymphoma may be a disorder of plasmacytoid cells in which case this raised plasma cell level may be of importance.

While an occasional prolymphocyte and an occasional Türk cell are seen in circulating blood in these cases of proven lymphoma, they were seen just as frequently in a control series of normal African children. This experience does not lead us to think that the finding of prolymphocytes in the circulating blood with a slight increase in the total lymphocyte count would enable us to predict the onset of lymphoma before the development of macroscopic tumours. This possibility has been raised by the studies by Bendixen (1958, 1959) of lymphocytic bovine leukosis in Denmark. Recent work in this field has been summarised by Steere (1959). This disease of young cattle has a geographically localised distribution depending on the movements of presumed infected animals. The infective agent is believed to be a filterable virus, there is a long incubation period, which explains the peculiar epidemiology of the disease, and there is ultimate involvement of the entire reticulo-endothelial system. This bovine disease has a marked superficial resemblance to the lymphomas seen in African children with respect to tumour morphology, cellular pattern and the distribution of " secondaries ". With increasing knowledge of this disease Bendixen was able to diagnose it by the method mentioned before the development of macroscopic tumours. Our attempt to show such changes in African children was unsuccessful but further investigations are necessary.

Since the commencement of these observations a number of children with lymphomas have been treated with Methotrexate (4-amino-$N^{10}$ methylpteroyl-glutamic acid). All the children treated showed a marked increase in the level of the circulating lymphocytes. The significance of this is unknown and is being further studied.

### SUMMARY

1. Circulating leucocyte and marrow cell counts were performed on a series of African children with lymphoma and compared with the most relevant published normal data.

2. No significant diagnostic variation of circulating leucocyte levels or morphology have been noted.

3. No significant variation from the expected myelogram pattern has been observed.

4. There is no evidence of a high level of circulating prolymphocytes and it is doubtful if a prognostic test can be devised.

5. Lymphoma in African children shows no blood or marrow features which could be considered as " leukaemic ".

My thanks are due to the Chief Medical Officer, Uganda, for permission to publish, to Professor J. N. P. Davies for help and encouragement and for access to the records of the Kampala Cancer Registry ; the Registry is maintained by the British Empire Cancer Campaign.

### REFERENCES

ALBRITTON, E. C. (Ed.).—(1952) ' Standard Values in Blood '. Philadelphia (Saunders).
BENDIXEN, H. J.—(1958) *Nord. VetMed.*, **10**, 273.—(1959) Report to 27th Session, Office Internationale des Epizooties, 516.
BLITSTEIN, I.—(1950) *Ann. Soc. belge. Méd. trop.*, **30**, 1401.
BURKITT, D. P.—(1958) *Brit. J. Surg.*, **46**, 218.
*Idem* AND O'CONOR, G. T.—(1961) *Cancer* (in press).
DACIE, J. V.—(1956) ' Practical Haematology '. London (Churchill).
DAVIES, A. G. M. AND DAVIES, J. N. P.—(1960) *Acta Un. int. Cancr.*, **16**, 1320.
DAVIES, J. N. P.—(1959) *In* ' Modern Trends in Pathology '. Ed. D. H. Collins. London (Butterworth).
LINHARD, L.—(1956) *Sang*, **27**, 142.
MOORE, R.—(1958) *J. trop. Med. (Hyg.)*, **61**, 70.
O'CONOR, G. T. AND DAVIES, J. N. P.—(1960) *J. Pediat.*, **56**, 526.
STEERE, J. L.—(1959) *Mod. Vet. Pract.*, **40**, 30.
VAN DEN BERGH, L. AND BLITSTEIN, I.—(1945) *Ann. Soc. belge. Méd. trop.*, **25**, 43.
O'CONOR, G. T.—(1961) *Cancer* (in press).